# High Mannose-Binding Lectin Serum Levels Are Associated with Diabetic Retinopathy in Chinese Patients with Type 2 Diabetes

**DOI:** 10.1371/journal.pone.0130665

**Published:** 2015-07-02

**Authors:** Qian Huang, Guilian Shang, Haohua Deng, Jie Liu, Yan Mei, Yancheng Xu

**Affiliations:** 1 Department of Endocrinology, Zhongnan Hospital, Wuhan University, Wuhan, Hubei province, P. R. China; 2 Department of Rheumatology, Tianyou Hospital, Wuhan University of Scienceand Technology, Wuhan, Hubei province, P.R. China; 3 School of Stomatology, Wuhan University, Wuhan, Hubei province, P.R. China; Centre for Cellular and Molecular Biology, INDIA

## Abstract

**Objective:**

To investigate mannose-binding lectin (MBL) serum levels in type 2 diabetic patients with and without diabetic retinopathy (DR).

**Methods:**

Serum MBL levels were determined in type 2 diabetic patients (N=324) as well as in 300 healthy control Subjects. Multivariate analyses were performed using logistic regression models. Receiver operating characteristic curves (ROC) was used to test the overall predict accuracy of MBL and other markers.

**Results:**

Diabetic patients with DR and vision-threatening diabetic retinopathy (VTDR) had significantly higher MBL levels on admission (P<0.0001 and P<0.0001). MBL improved the area under the receiver operating characteristic curve of the diabetes duration for DRfrom 0.82(95% confidence interval [CI], 0.77–0.86) to 0.88(95% CI, 0.82–0.96; P<0.01) and for VDTR from 0.85(95% CI, 0.77–0.92) to 0.90(95% CI, 0.83–0.96; P<0.01). Multivariate logistic regression analysis adjusted for common risk factors showed that serum MBL levels(per log-unit increase) was an independent predictor of DR (OR=3.45; 95%CI: 1.42–7.05) and VTDR (OR=4.42; 95%CI: 1.51-8.18).

**Conclusion:**

MBL is a novel, independent diagnostic marker of DR in type 2 diabetic patients, suggesting that MBL may be involved in the pathogenesis of DR in diabetic patients.

## Introduction

Type 2 diabetes (T2DM) has become a major public health problem in China. In 2009, the age-standardized prevalences of total diabetes and prediabetes were 9.7% and 15.5%, respectively, accounting for 92.4 million adults with diabetes and 148.2 million adults with prediabetes [1]. Diabetic retinopathy (DR) is the most frequent cause of new cases of blindness among adults aged 20–74 years[2]. Approximately 29% of U.S. adults with type 2 diabetes have DR [3]. Liu et al [4] reported that 14.8% Chinese patient with T2DM have DR.

The presence of DR was associated with an increased risk of all-cause mortality and cardiovascular events in diabetic patient [5]. Up to 21% of patients with T2DM have retinopathy at the time of first diagnosis of diabetes, and most develop some degree of retinopathy over time. Vision loss due to diabetic retinopathy results from several mechanisms and central vision may be impaired by macular edema or capillary nonperfusion[6]. Although the major risk factors for DR (e.g., hyperglycemia, hypertension, dyslipidemia) have been examined in many epidemiologic studies and clinical trials, there is considerable variation inthe consistency, pattern, and strength of these risk factors [3].

Mannose-binding lectin (MBL) is synthesized by hepatocytes and belongs to the family of C-type lectins[7]. MBL is a part of the complement cascadeand plays an important role in the first line of defense ofthe innate immune system against pathogenicmicroorganisms [8]. MBL exerts an important role in the innate immune system [9], and several studies have indicated that low levels of MBL affect the outcome of kidney graft survival [10], infectious diseases [11], critical illness [12] and neonatal sepsis and pneumonia [13]. Interestingly, high levels of MBL offer protection against invading microorganisms and may in other situations confer biological disadvantages [14]. MBL may aggravate local and systemic inflammation throughcomplement activation and modulation of proinflammatory cytokine production [15]. Previous studies have suggested that increased MBL levels are associated with an increased risk of stroke and poor functional outcomes after stroke in Chinese population [16–17].

There have been great controversies in studying relationship between MBL and diabeticcomplications. Siezenga et al. [18] found that log MBL levels were not associated with the occurrence of cardiovascular events in type 2 diabetic South Asians, while Elawa et al [19] reported that elevated serum MBL in T2DM patients indicated a possible poor diabetic control and bad progression of the disease with possibility of the presence. Consistent with this, Bouwman et al. [20]concluded that MBL serum concentration and complex activity are increased in early-onset diabetic patients upon manifestation independently of genetic predisposition to high MBL production, indicating a possible role in the immunopathogenesis of type 1 diabetes. In addition, Hansen et al [21] found that the median MBL concentration was higher in type 1 diabetic patients than in healthy controls. However, no data are available on the role of MBL in the progression of DR in Chinese patients with T2DM. In this study, we therefore evaluated serum MBL levels in Chinese type 2 diabetic patients with and without diabetic retinopathy.

## Method

We conducted a prospective cohort study at the endocrinology department of our hospital. We consecutively recruited 324 Chinese with type 2 diabetes aged 31–74 years between October 2012 and May 2014. Participants were excluded if they had a history of epilepsy or glaucoma, had undergone previous vitreal surgery, and/or had a cataract on examination. Participants who had no light perception or severe visual impairment in both eyes or had a severe infection in one or both eyes were excluded. A group of 300 age-matched healthy subjects served as control subjects. The study followed the tenets of the Declaration of Helsinki and was approved by the Institute ethics committee of Zhongnan Hospital, Wuhan University, with written informed consent obtained from each participant.

We used the Canon CR6-45NM ophthalmic digital imaging system and Canon EOS 10D digitalcamera (Canon, Tokyo, Japan) to take 2 digital images per eye through a nonpharmacologicallydilatedpupil. DR was defined as the presence of 1 or more retinal microaneurysms or retinal blot hemorrhages with or without more severe lesions (hard exudates, soft exudates, intraretinalmicrovascular abnormalities, venous beading, retinal new vessels, preretinal and vitreous hemorrhage, and fibroproliferans) using the Early Treatment Diabetic Retinopathy Study (ETDRS) grading standards[22]. DR severity was categorized as non-proliferative diabetic retinopathy (NPDR; level 20 through level 53) and proliferative diabetic retinopathy (PDR; level≥60). Diabetic macular edema (DME) was defined as present or absent and classified as with or without clinically significant macular edema; and vision-threatening diabetic retinopathy (VTDR) was defined as the presence of PDR and/or DME. T2DM was defined as self-report of a previous diagnosis of the disease by a clinician (excluding gestational diabetes mellitus) or hemoglobin A1c (HbA1c) of 6.5% or greater [23]. At baseline, we requested individual participant data regarding presence and severity of DR, DME status, age, sex, ethnicity, diabetes type and duration, HbA1c, systolic and diastolic blood pressure, cigarette smoking status, BMI, and current use of diabetes, antihypertensive, and lipid-lowering medications.

Blood samples of patients and controls were obtained at 7:00 AM in the next morning of the day of inclusion under fasting state. 2 ml of blood were placed into a dry clean tube and left to clot at room temperature, and then separated by centrifugation for 15 min. Clotted blood was centrifuged within 1h and serum stored at -80°C. HbA1c was measured by high-performance liquid chromatography (HLC-723 G7; TOSHO, Japan) with a normal range of 4–6%. MBL was measured by time-resolved immune-fluorometricassay on serum samples. Microwells coated with anti-MBL antibody were incubated with dilutions of patient serum, were developed with europium-labelled anti-MBL antibody, and europium was quantified with time-resolved fluorometric assay (Baoman Biological Technology Co., Ltd, Shanghai, China). The detection limit was 1.8ug/L. The standard concentrations in these kits range from 1.8 to 100ug/L, providing a range of 180–10000ug/L at 1/100 dilution. The coefficients of variation (CV) for the intra- and inter-assay reproducibility are 4.0–5.8% and 6.7–9.4%, respectively.

Results are expressed as percentages for categorical variables and as medians (interquartile ranges, IQRs) for the continuous variables. Univariate data on demographic and clinical features were compared by Mann-Whitney U-test or Chi-Square test as appropriate. Correlations among continuous variables were assessed by the Spearman rank-correlation coefficient. To investigate whether MBL allows predicting of both DR and VTDR in diabetes different statistical methods were used. First, the relation of MBL with the two points was investigated with the use of logistic regression models. Therefore, common logarithmic transformation (ie, base 10) was performed to obtain normal distribution for skewed variables (ie, MBL concentrations) as the resulting model yielded smaller Akaike Information Criterion, which was chosen to compare the results. We used crude models and multivariate models adjusted for all significant predictors and report odds ratios (ORs). For multivariate analysis, we included confounders, known risk factors, and other predictors as assessed in univariate analysis. Second, receiver operating characteristic curves (ROC) was used to test the overall predict accuracy of MBL and other markers, and results were reported as area under the curve (AUC). All statistical analysis was performed with SPSS for Windows, version 20.0 (SPSS Inc., Chicago, IL, USA)and the ROCR package (version 1.0–2), which is available from CRAN repository (http://cran.r-project.org/). Statistical significance was defined as P< 0.05.

## Results

### Patient Characteristics

There were 324 people with type 2 diabetes eligible for the study. The median age of patients included in this study was 55(IQR, 42–68) years and 55.6% were men. The median time of diabetes duration was 7.5 (IQR, 6.0–10.0) years. DR was found in 115 patients (35.5%). Forty-one patients were defined as VTDR, thus the rate was 12.7%. Basal characteristics of those patients were provided in table 1.

**Table 1:** Basal characteristic of diabetes patients with DR or without DR.

	Diabetes	Retinopathy status		
Characteristics	N = 324	Yes(N = 115)	No(N = 209)	P
Age at baseline (IQR, years)	55(42–68)	56(42–68)	55(43–67)	NS
Male (%)	55.6	52.2	57.4	NS
Diabetes duration (IQR, years)	7.5(6.0–10)	9.0(8.0–12.5)	6.0(4.5–8.0)	<0.001
BMI (IQR, kg/m2)	29.1(26.5–31.4)	29.5(26.9–31.8)	28.4(25.6–31.1)	NS
Systolic blood pressure (IQR, mmHg)	134(127–144)	145(132–150)	122(115–135)	<0.01
Smoking status (%)	40.1	39.1	40.7	NS
Current alcohol intake (%)	34.6	34.8	34.4	NS
Intensive glucosetreatment (%)	41.7	47.8	38.3	<0.01
Blood pressuretreatment (%)	37.7	38.3	37.3	NS
Use of lipid-lowering medication(%)	33.3	32.2	33.9	NS
Laboratory findings(IQR)				
HbA1c (%)	7.9(7.1–8.7)	8.5(7.6–9.7)	7.2(6.4–8.1)	<0.001
Serum creatinine (umol/L)	92(75–100)	94(77–103)	87(74–97)	NS
Total cholesterol (mmol/L)	4.7(3.9–5.5)	4.9(4.2–5.7)	4.4(3.7–5.2)	<0.01
Triglycerides (mmol/L)	1.5(0.9–1.8)	1.6(1.0–1.9)	1.4(0.9–1.7)	NS
LDL-cholesterol (mmol/l)	2.6(1.9–3.0)	2.6(2.1–3.1)	2.5(1.9–2.9)	NS
HDL-cholesterol (mmol/l)	1.5(1.3–1.7)	1.6(1.4–1.8)	1.5(1.2–1.7)	NS
Hs-CRP(mg/dL)	0.98(0.44–2.10)	1.48(0.62–3.01)	0.62(0.32–1.57)	<0.001
MBL(ug/L)	2976(2421–3521)	3388(2942–4080)	2653(2157–3110)	<0.0001
Any DR (%)	35.5	—	—	—
PDR	8.0			
DME	9.3			
VTDR	12.7			

Results are expressed as percentages or as medians (IQR); BMI, body mass index; Hs-CRP, High-sensitivity- C-reactive protein; DR, diabetic retinopathy; PDR, proliferative diabetic retinopathy; DME, diabetic macular edema; VTDR; vision-threatening diabetic retinopathy.

### MBL and Clinical Varies

**Fig 1:**
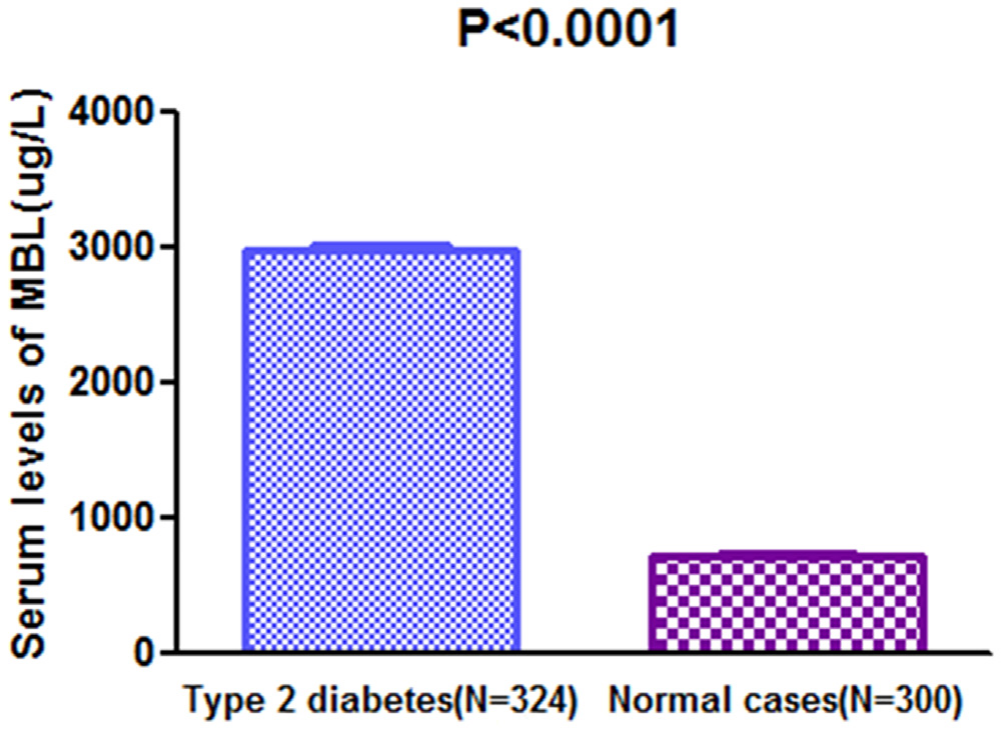
A separate histogram of serum MBL levels in diabetic patientsand normal controls. The horizontal lines in the top indicate mean levels.*P*values refer to Mann-Whitney *U* tests for differencesbetween groups.

**Fig 2:**
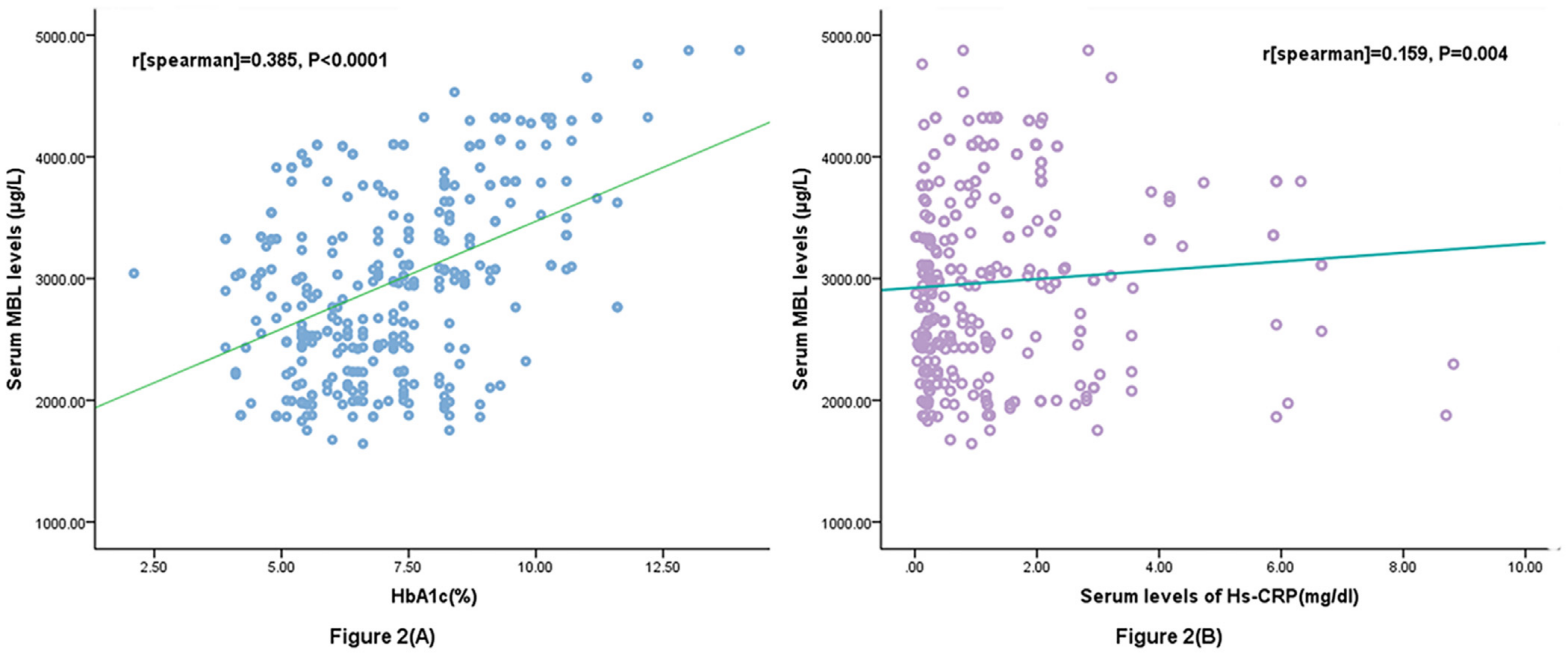
Correlation between the serum MBL levels and other factors (a) Correlation between the serum MBL levels and HbA1c; (b) Correlation between the serum MBL levels and Hs-CRP.

The results indicated that the serum MBL levels were significantly (P<0.0001) higher in diabetes patients as compared to normal cases [2976(IQR, 2421–3521μg/L); 732(IQR, 576–894μg/L), respectively; Fig 1]. There was a modest positive correlation between levels of MBL and HbA1c (r = 0.385, P<0.0001; Fig 2A). In addition, there was a significant, albeit weak, positive correlation between MBL levels and Hs-CRP (r = 0.159, P = 0.004; Fig 2B). Furthermore, there was no correlation between serumlevels of MBL and others factors, such as, sex, age, creatinine, triglyceride, cholesterol, LDL and HDL, duration of diabetes, or daily insulin dose(P>0.05).

### MBL and DR

**Fig 3:**
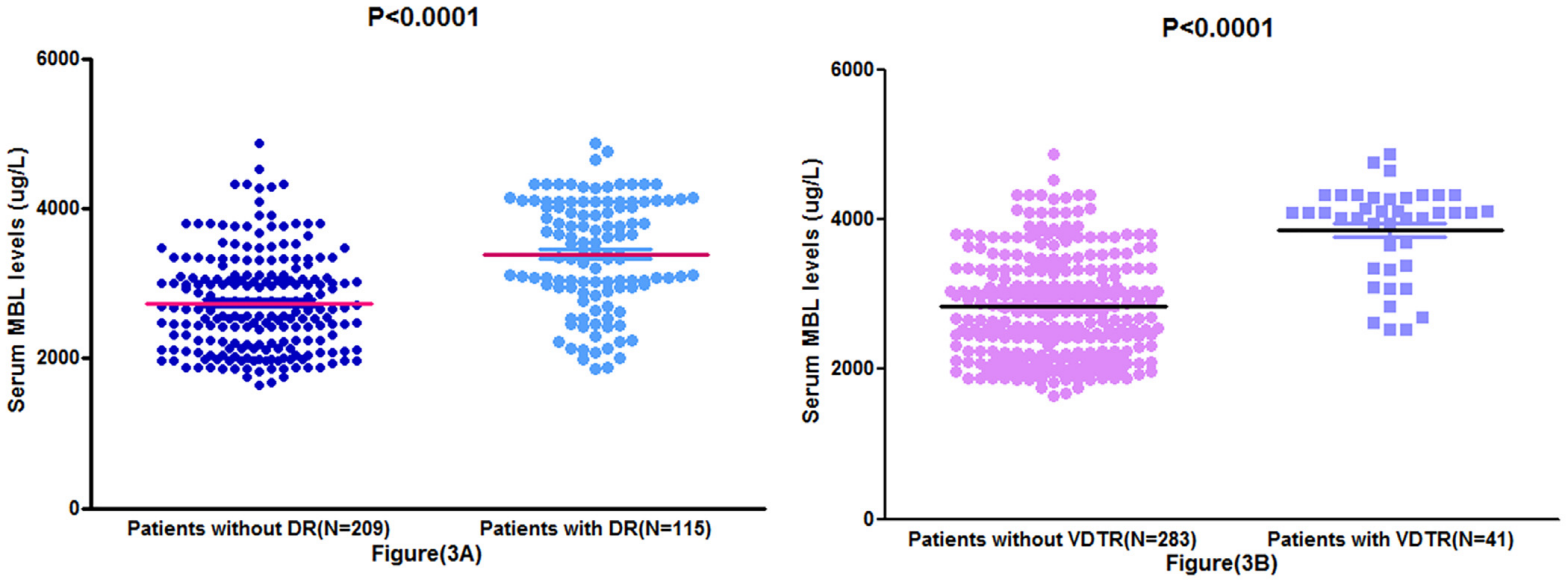
Distribution of serum MBL levels in diabetic patients with different groups. The horizontal lines indicate mean levels. (A) Distribution of serum MBL levels in diabetic patients with DR and without DR; (B) Distribution of serum MBL levels in diabetic patients withvision-threatening diabetic retinopathy (VTDR) and without VTDR. P values refer to Mann-Whitney U tests for differences between groups.

In the 115 patients with DR, serum MBL levels were higher compared with those in patients without DR [3388(IQR, 2942–4080) μg/L vs. 2653(IQR, 2157–3110) μg/L; P<0.0001; Fig 3A). In univariate logistic regression analysis, we calculated the odds ratio (OR) of log-transformed MBL levels as compared with other risk factors as presented in Table 2. With an unadjusted OR of 7.12 (95% CI, 3.81–13.15), MBL had a strong association with DR. After adjusting for all other significant predictors, MBL remained can be seen as an independent DR predictor with an adjusted OR of 3.45 (95% CI, 1.42–7.05; P<0.0001). In multivariate analysis, there was an increased risk of DR associated with MBL levels≥3521ug/L(3^rd^quartiles; OR 3.10, 95% CI: 1.72–5.48; P<0.0001) after adjusting for possible confounders. In addition, male sex, diabetes duration, HbA1c, Hs-CRP, intensive glucose treatment and systolic BP were also can be seen as DR predictors in multivariate analysis (Table 2).

**Table 2:** Univariate and multivariate logistic regression analysis for DR and VTDR.

Parameter	Univariate Analysis			Multivariate Analysis		
	OR ^a^	95% CI ^a^	*P*	OR ^a^	95% CI ^a^	*P*
Predictor: DR						
MBL^b^	7.12	3.81–13.15	<0.0001	3.45	1.42–7.05	<0.0001
MBL(≥3^rd^quartiles) ^c^	3.92	2.31–6.56	<0.0001	3.10	1.72–5.48	<0.0001
Male sex	1.20	1.05–1.38	0.005	1.15	1.06–1.28	0.002
HbA1c	1.08	1.03–1.20	<0.001	1.05	1.01–1.16	<0.001
Diabetes duration	1.24	1.13–1.30	<0.0001	1.16	1.10–1.24	<0.0001
Hs-CRP	1.10	1.04–1.18	<0.001	1.08	1.03–1.18	<0.001
Intensive glucosetreatment	2.03	1.25–3.45	0.018	1.90	0.91–3.15	0.311
Hypertension	1.58	1.31–1.82	0.009	1.30	1.12–1.44	0.011
Predictor: VTDR						
MBL ^b^	9.14	3.16–1.8.22	<0.0001	4.42	1.51–8.18	<0.0001
MBL(≥3^rd^quartiles)^c^	9.55	4.51–19.78	<0.0001	7.83	3.35–18.31	<0.0001
Male sex	1.16	1.06–1.46	0.031	1.08	1.02–1.35	0.037
HbA1c	1.12	1.04–1.33	<0.001	1.08	1.03–1.16	<0.001
Diabetes duration	1.22	1.08–1.36	<0.0001	1.12	1.04–1.25	<0.0001
Hs-CRP	1.14	1.06–126	0.003	1.09	1.03–1.18	0.006
Intensive glucosetreatment	1.22	1.09–1.54	<0.001	1.14	1.06–1.28	0.003
Hypertension	1.71	1.27–3.01	0.009	1.58	1.28–2.30	0.006

^a^Note that the odds ratio corresponds to a unit increase in the explanatory variable.

^b^log-transformed, note that the odds ratio corresponds to a log-unit increase in the explanatory variable

^c^ MBL(≥3^rd^quartiles) as one predictor in the multivariate logistic regression analysis

OR, odds ratio; CI, confidence interval; Hs-CRP, High-sensitivity- C-reactive protein;DR, diabetic retinopathy; VTDR; vision-threatening diabetic retinopathy.

**Fig 4:**
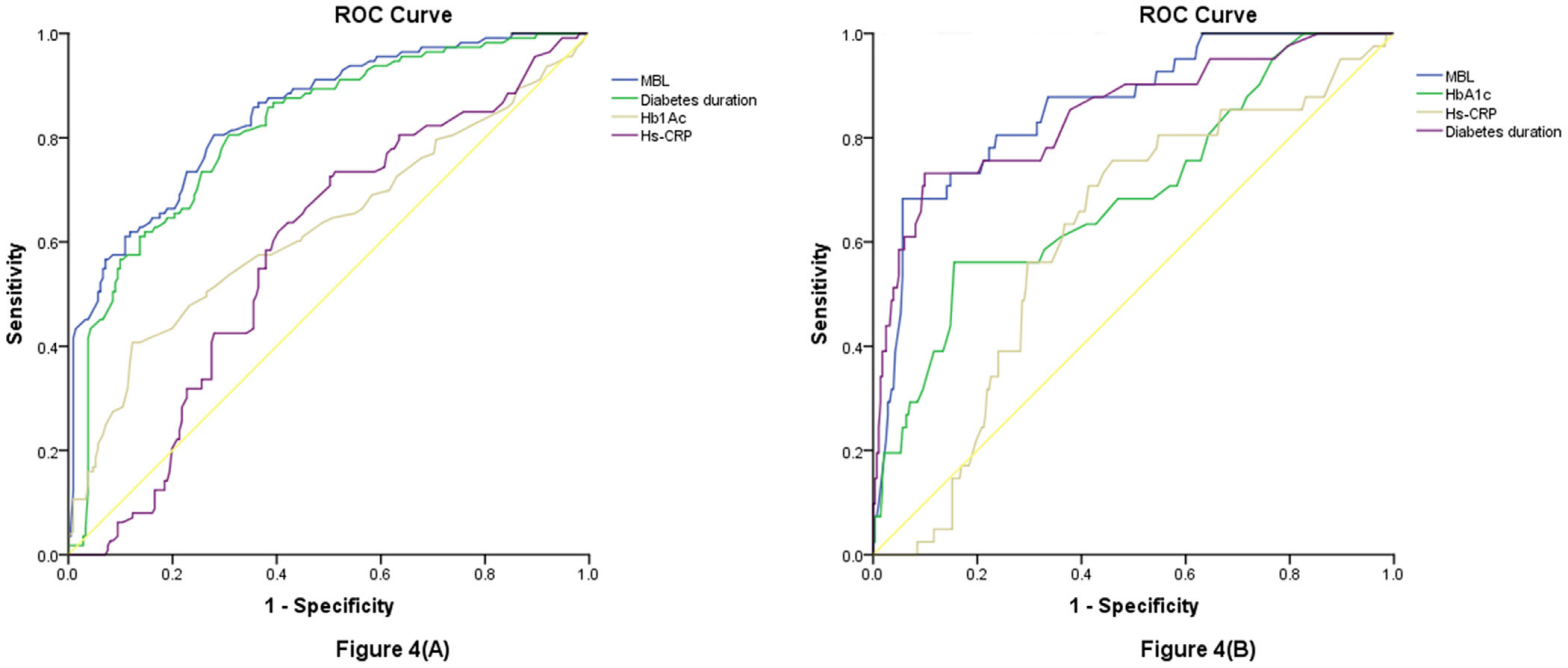
Receiver operating characteristic (ROC) curves were utilized to evaluate the accuracy of markers to diagnoseDR or VDTR. (A) Receiver operator characteristic curve demonstrating sensitivity as a function of 1-specificity for diagnosing the DR based on the MBL, Hs-CRP, HbA1c anddiabetes duration; (B) Receiver operator characteristic curve demonstrating sensitivity as a function of 1-specificity for diagnosing the VDTR based on the MBL, Hs-CRP, HbA1c anddiabetes duration.

With an AUC of 0.84 (95% CI, 0.80–0.89), MBL showed a significantly greater discriminatory ability to diagnose DR as compared with Hs-CRP (AUC, 0.58; 95% CI, 0.52–0.65; P<0.0001), HbA1c (AUC, 0.63; 95% CI, 0.56–0.70; P<0.001) and age (AUC, 0.55; 95% CI, 0.49–0.63; P<0.0001), while was in the range of diabetes duration (AUC, 0.82; 95% CI, 0.77–0.86; P = 0.056; Fig 4A). Interestingly, MBL improved the ability of diabetes duration to diagnose DR (AUC of the combined model, 0.88; 95% CI, 0.82–0.96; P<0.01). This improvement was stable in an internal 5-fold cross validation that resulted in an average AUC (standard error) of 0.82 (0.030) for the diabetes duration and 0.88(0.021) for the combined model, corresponding to a difference of 0.06(0.009). Table 3.

**Table 3:** Receiver operating characteristics curve analysis.

Parameter	DR			VTDR		
	AUC	95% CI	*P*	AUC	95% CI	*P*
MBL	0.84	0.80–0.89		0.86	0.80–0.92	
Age	0.55	0.52–0.65	<0.0001	0.59	0.53–0.64	<0.0001
Male	0.57	0.54–0.64	<0.0001	0.60	0.54–0.66	<0.0001
Diabetes duration	0.82	0.77–0.86	0.056	0.85	0.77–0.92	0.126
Systolic blood pressure	0.60	0.55–0.66	<0.0001	0.62	0.57–0.69	<0.0001
HbA1c	0.63	0.56–0.70	<0.0001	0.69	0.60–0.78	<0.0001
Hs-CRP	0.58	0.52–0.65	<0.0001	0.61	0.53–0.70	<0.0001
Combined model^a^	0.88	0.82–0.96	<0.01	0.90	0.83–0.96	<0.01

AUC, areaunder the curve;CI, confidence interval; OR, odds ratio; Hs-CRP, High-sensitivity- C-reactive protein; DR, diabetic retinopathy; VTDR; vision-threatening diabetic retinopathy.

^a^Combined model = MBL and diabetes duration

### MBL and VTDR

In the 41 patients with VTDR, serum MBL levels were higher compared with those in patients without DR [4087(IQR, 3360–4290) μg/L vs. 2852(IQR, 2234–3325) μg/L; p<0.0001; Fig 3B). In univariate logistic regression analysis, we calculated the odds ratio (OR) of log-transformed MBL levels as compared with other risk factors as presented in Table 2. In multivariate analysis, after adjusting for all other significant predictors, MBL remained can be seen as an independent DR predictor with an adjusted OR of 4.42 (95% CI, 1.51–8.18; P<0.0001). In addition, there was an increased risk of DR associated with MBL levels≥3521ug/L(3^rd^quartiles; OR 7.89, 95% CI: 3.42–18.53; P<0.0001) after adjusting for possible confounders. In addition, male sex, diabetes duration, HbA1c, Hs-CRP and systolic BP were also can be seen as DR predictors in multivariate analysis (Table 2).

With an AUC of 0.86 (95% CI, 0.80–0.92), MBL showed a significantly greater discriminatory ability to diagnose VTDR as compared with Hs-CRP (AUC, 0.61; 95% CI, 0.53–0.70; P<0.0001), HbA1c (AUC, 0.69; 95% CI, 0.60–0.78; P<0.0001) and age (AUC, 0.59; 95% CI, 0.53–0.64; P<0.001), while was in the range of diabetes duration (AUC, 0.85; 95% CI, 0.77–0.92; P = 0.126; Fig 4B). Again, MBL improved the ability of diabetes duration to diagnose VTDR (AUC of the combined model, 0.90; 95% CI, 0.83–0.96; P<0.01). This improvement was also stable in an internal 5-fold cross validation that resulted in an average AUC (standard error) of 0.85 (0.022) for the diabetes duration and 0.90 (0.014) for the combined model, corresponding to a difference of 0.05(0.008; Table 3).

## Discussions

Several studies have shown that deficiency of MBL increases the overall susceptibility of an individual to infectious disease [24]. The most striking example of this is the association of acute respiratory tract infections with MBL deficiency in early childhood [25]. Clinical studies have shown that MBL insufficiency is associated with bacterial infection in patients with neutropenia and meningococcal sepsis. Numerous other potential infectious disease associations have been described [26]. In contrast, there is evidence that for some intracellular parasites MBL deficiency may be protective and this might explain the high frequency of *MBL* mutations in sub-Saharan Africa and South America [24]. MBL is an example of a pattern recognition molecule that plays a dual role in modifying inflammatory responses to sterile and infectious injury [27].

Previous studies had suggested that there may be a link between complement activation and the development of diabetic complications [28–29]. Mounting evidence supports the importance of the MBL pathway of complement activation in innate immunity [30]. One study suggested that MBL and the lectin complement pathway play a significant role in vascular dysfunction and cardiomyopathy after acute hyperglycemia [31]. In this study, we firstly assessed the serum MBL levels with regard to their accuracy to predict DR and VTDR in patients with T2DM in Chinese sample. Consistent with our findings, Man et al. [32] also found that evaluated serum levels of MBL can be seen as an independent marker of DR even after correcting for possible confounding factors in type 2 diabetic patients.

In previous studies, Hansen et al. [21] demonstrated that circulating MBL concentrations are significantly elevated in patients with type 1 diabetes and suggested a possible role of MBL in the pathogenesis of renovascular complications in diabetes. In another study, they also found that MBL may be involved in the pathogenesis of micro-and macrovascular complications in type 1 diabetes [15]. In this study, we confirmed that of elevated MBL were correlated with DR and VTDR, and added significant additional predictive information to the diabetes duration, suggesting a possible role of MBL in the pathogenesis of DR complications in T2DM. Similarly, Hansen et al. [33] reported that in patients with type 2 diabetes, measurements of MBL alone or in combination with CRP can provide prognostic information on mortality and the development of albuminuria.

The importance of glycaemia, blood pressure and diabetes duration as risk factors for DR is already well established [34]. Male sex has also been reported as a risk factor in other studies [35]. Importantly, in our study, we found that MBL was a risk factor for DR. In addition, male sex, diabetes duration, HbA1c, Hs-CRP and systolic BP was also reported.

High CRP and MBL levels could both be a sign ofan inflammatory state, and MBL is a slower-reacting and muchweaker acute-phase reactant than CRP [17]. Navarro et al[36] found that inflammatory parameters(Hs-CRP) in patients with type 2 diabetes at an early stage of nephropathy are independently associated with urinary albumin excretion (UAE). We did indeed observesignificantly higher Hs-CRP levels among patientswith diabetic retinopathy compared with others. However, the relationship between MBL levelsand diabetic retinopathy persisted on additional adjustment for Hs-CRP, which indicates thatCRP and MBL may carry different types of information asmarkers of inflammation.

In a meta- analysis, a total of 35 studies (1980–2008) provided data from 22,896 individuals with diabetes, and the overall prevalence was 34.6% for any DR [3]. Similarly, in our study, 35.5% of the diabetes patients had DR. Our findings are in line with reports from recent population studies in which the prevalence of DR ranged from 6% to 33% [33, 37–38]. Zhang et al. [39]reported that the estimated prevalence of DR and VDTR was 28.5% (95% confidence interval [CI], 24.9%-32.5%) and 4.4% (95% CI, 3.5%-5.7%) among US adults with diabetes, respectively. Differences in study methodologies, population characteristics, and ascertainment and classification of DR have made direct comparisons between studies difficult.

Despite extensive research, the exact pathogenesis of DR is still unknown. Whether higher serum MBL level was acause of or merely a marker for DR in diabetesremain uncertain. MBL is more likely to be acontributing factor to the DR rather than a mere marker, and may involve multiple mechanisms. Firstly, MBL is a slower-reacting and much weaker acute-phase reactant than CRP [12], but it is possible that the differences in MBL concentrations between patients with and without DR may reflect differences in inflammatory activity. In addition, MBLmay aggravate local and systemic inflammation throughcomplement activation [40] and modulation of proinflammatorycytokine production [41]. We can speculate that high levels of MBL and subsequent complement activation will result in a net proinflammatory state, potentiating allograft damage, leading downstream to chronic allograft dysfunction. It could thus be hypothesized that in diabetic patients, high levels of MBL may contribute to the development of DR through aggravated complement activation. Thirdly, oxidative stress leading to changes in cell surfaceglycosylations may activate the complement system viaMBL [40], and MBL binding to fructoselysine and the ensuing complement activation may provide a physiopathological link between enhanced glycation and complement activation in diabetes [42]. Interestingly, we found that there was a modest positive correlation between levels of MBL and HBA1c (P<0.0001).

A number of issues have to be taken into account wheninterpreting the results of the present study. Firstly, without serial measurement of the circulating MBL, this study yielded no data regarding when and how long of MBL was elevated in these patients. Additionally, it should be investigated whether serial MBL testing further improves the risk stratification of these patients. Secondly, the samples were also geographically limited, potentially limiting the generalizability of our results. Larger studies are needed to confirm our results and elucidate the underlying mechanisms. Thirdly, MBL genotypes were not determined. Those results should be useful to explain the differences MBL concentration between studies. Lastly, this was only a preliminary study; further studies should investigate whether MBL can help physicians tailor the therapy in view of the relative risk and allocate resources accordingly and whether this strategy might affect DR outcome.

## Conclusions

The present study demonstrated that serum MBL level was an independent risk factor for DR and VDTR in Chinese patients with T2DM, suggesting a possible role of MBL in the pathogenesis of DR complications. We suggested that further studies should be carried out with respect to what was the cause of the increased MBL levels and the role in the pathology of the DR.

## References

[1] Yang W, Lu J, Weng J, Jia W, Ji L, Xiao J, et al Prevalence of diabetes among men and women in China. New England Journal of Medicine. 2010; 362: 1090–1101. 10.1056/NEJMoa0908292 20335585

[2] Klein BE. Overview of epidemiologic studies of diabetic retinopathy. Ophthalmic Epidemiol. 2007; 14: 179–183. 17896294 10.1080/09286580701396720

[3] Yau JWY, Rogers SL, Kawasaki R, Lamoureux EL, Kowalski JW, Bek T, et al Global prevalence and major risk factors of diabetic retinopathy. Diabetes care. 2012; 35:556–564. 10.2337/dc11-1909 22301125 PMC3322721

[4] Liu Z, Fu C, Wang W, Xu B. Rersearch prevalence of chronic complications of type 2 diabetes mellitus in outpatients–a cross sectional hospital based survey in urban China. Health Qual Life Outcomes. 2010; 8: 62 10.1186/1477-7525-8-62 20579389 PMC2906445

[5] Kramer CK, Rodrigues TC, Canani LH, Gross JL, Azevedo MJ. Diabetic retinopathy predicts all-cause mortality and cardiovascular events in both type 1 and 2 diabetes meta-analysis of observational studies. Diabetes Care. 2011; 34: 1238–1244. 10.2337/dc11-0079 21525504 PMC3114518

[6] Fong DS, Aiello L, Gardner TW, King GL, Blankenship G, Cavallerano JD, et al Diabetic retinopathy. Diabetes Care. 2003; 26:S99–S102. 12502630 10.2337/diacare.26.2007.s99

[7] Chang WC, White MR, Moyo P, McClear S, Thiel S, Hartshorn KL, et al Lack of the pattern recognition molecule mannose-binding lectin increases susceptibility to influenza A virus infection. BMC immunology. 2010; 11: 64 10.1186/1471-2172-11-64 21182784 PMC3022599

[8] Keller TT, van Leuven SI, Meuwese MC, Wareham NJ, Luben R, Stroes ES, et al Serum levels of mannose-binding lectin and the risk of future coronary artery disease in apparently healthy men and women. Arterioscler Thromb Vasc Biol. 2006; 26:2345–2350. 16902159 10.1161/01.ATV.0000240517.69201.77

[9] Turner MW. Mannose-binding lectin: the pluripotent molecule of the innate immune system. Immunology today. 1996; 17: 532–540. 8961631 10.1016/0167-5699(96)10062-1

[10] Bay JT, Sørensen SS, Hansen JM, Madsen HO, Garred P. Low mannose-binding lectin serum levels are associated with reduced kidney graft survival. Kidney international. 2012; 83: 264–271. 10.1038/ki.2012.373 23172101

[11] Eisen D P, Minchinton R M. Impact of mannose-binding lectin on susceptibility to infectious diseases. Clinical Infectious Diseases. 2003; 37: 1496–1505. 14614673 10.1086/379324

[12] Hansen TK, Thiel S, Wouters PJ, Christiansen JS, Van den Berghe G. Intensive insulin therapy exerts antiinflammatory effects in critically ill patients and counteracts the adverse effect of low mannose-binding lectin levels. J Clin Endocrinol Metab.2003; 88:1082–1088. 12629088 10.1210/jc.2002-021478

[13] Özkan H, Köksal N, Cetinkaya M, Kiliç Ş, Çelebi S, Oral B, et al Serum mannose-binding lectin (MBL) gene polymorphism and low MBL levels are associated with neonatal sepsis and pneumonia. Journal of Perinatology. 2011; 32: 210–217. 10.1038/jp.2011.79 21681178

[14] Ezekowitz RA. Genetic heterogeneity of mannose-binding proteins: the Jekyll and Hyde of innate immunity? Am J Hum Genet. 1998; 62:6–9. 9443889 10.1086/301696 PMC1376820

[15] Hansen TK, Tarnow L, Thiel S, Steffensen R, Stehouwer CD, Schalkwijk CG, et al Association between mannose-binding lectin and vascular complications in type 1 diabetes. Diabetes. 2004; 53:1570–1576. 15161763 10.2337/diabetes.53.6.1570

[16] Wang ZY, Sun ZR, Zhang LM. The relationship between serum mannose-binding lectin levels and acute ischemic stroke risk. Neurochem Res. 2014; 39: 248–253. 10.1007/s11064-013-1214-x 24309995

[17] Zhang ZG, Wang C, Wang J, Zhang Z, Yang YL, Gao L, et al Prognostic Value of Mannose-Binding Lectin: 90-Day Outcome in Patients with Acute Ischemic Stroke. Mol Neurobiol. 2015; 51:230–239. 10.1007/s12035-014-8682-0 24691546

[18] Siezenga MA, Shaw PK, Daha MR, Rabelink TJ, Berger SP. Low Mannose-Binding Lectin (MBL) genotype is associated with future cardiovascular events in type 2 diabetic South Asians. A prospective cohort study. Cardiovasc Diabetol. 2011; 10:60 10.1186/1475-2840-10-60 21729275 PMC3157421

[19] Elawa G, AoudAllah AM, Hasaneen AE, El-Hammady AM. The predictive value of serum mannan-binding lectin levels for diabetic control and renal complications in type 2 diabetic patients. Saudi Med J. 2011; 32:784–790. 21858386

[20] Bouwman LH, Eerligh P, Terpstra OT, Daha MR, de Knijff P, Ballieux BE, et al Elevated levels of mannose-binding lectin at clinical manifestation of type 1 diabetes in juveniles. Diabetes. 2005; 54: 3002–3006. 16186405 10.2337/diabetes.54.10.3002

[21] Hansen TK, Thiel S, Knudsen ST, Gravholt CH, Christiansen JS, Mogensen CE, et al Elevated levels of mannan-binding lectin in patients with type 1 diabetes. J Clin Endocrinol Metab. 2003; 88: 4857–4861. 14557465 10.1210/jc.2003-030742

[22] Early Treatment Diabetic Retinopathy Study Research Group. Grading diabetic retinopathy from stereoscopic color fundus photographs—an extension of the modified Airlie House classification: ETDRS report number 10: Early Treatment Diabetic Retinopathy Study Research Group. Ophthalmology. 1991; 98:S786–S806. 2062513

[23] AmericanDiabetes Association. Diagnosis and classification of diabetes mellitus. Diabetes Care. 2010; 33:S62–S69. 10.2337/dc10-S062 20042775 PMC2797383

[24] Turner MW. The role of mannose-binding lectin in health and disease. Molecular immunology. 2003; 40: 423–429. 14568388 10.1016/s0161-5890(03)00155-x

[25] Koch A, Melbye M, Sørensen P, Homøe P, Madsen HO, Mølbak K, et al Acute respiratory tract infections and mannose-binding lectin insufficiency during early childhood. JAMA. 2001; 285: 1316–1321. 11255386 10.1001/jama.285.10.1316

[26] Eisen DP, Minchinton RM. Impact of mannose-binding lectin on susceptibility to infectious diseases. Clinical Infectious Diseases. 2003; 37: 1496–1505. 14614673 10.1086/379324

[27] Walsh MC, Bourcier T, Takahashi K, Shi L, Busche MN, Rother RP, et al Mannose-binding lectin is a regulator of inflammation that accompanies myocardial ischemia and reperfusion injury. The Journal of Immunology. 2005; 175: 541–546. 15972690 10.4049/jimmunol.175.1.541

[28] Acosta J, Hettinga J, Fluckiger R, Krumrei N, Goldfine A, Angarita L, et al Molecular basis for a link between complement and the vascular complications of diabetes. Proc Natl Acad Sci U S A 2000; 97:5450–5455. 10805801 10.1073/pnas.97.10.5450 PMC25849

[29] Hsu SI, Couser WG. Chronic progression of tubulointerstitial damage in proteinuric renal disease is mediated by complement activation: a therapeutic role for complement inhibitors? J Am Soc Nephrol. 2003; 14:S186–S191. 12819326 10.1097/01.asn.0000070032.58017.20

[30] Vang Petersen S, Thiel S, Jensenius JC. The mannan-binding lectin pathway of complement activation: biology and disease association. Molecular immunology. 2001; 38: 133–149. 11532276 10.1016/s0161-5890(01)00038-4

[31] Pavlov VI, La Bonte LR, Baldwin WM, Markiewski MM, Lambris JD, Stahl GL. Absence of mannose-binding lectin prevents hyperglycemic cardiovascular complications. The American journal of pathology. 2012 180: 104–112. 10.1016/j.ajpath.2011.09.026 22079428 PMC3338344

[32] Man X, Zhang H, Yu H, Ma L, Du J. Increased serum mannose binding lectin levels are associated with diabetic retinopathy. Journal of diabetes and its complications. 2015; 29: 55–58. 10.1016/j.jdiacomp.2014.09.013 25457461

[33] Hansen T K, Gall M A, Tarnow L, Thiel S, Stehouwer CD, Schalkwijk CG, et al Mannose-binding lectin and mortality in type 2 diabetes. Archives of internal medicine. 2006; 166: 2007–2013. 17030835 10.1001/archinte.166.18.2007

[34] Looker H C, Nyangoma S O, Cromie D, Olson JA, Leese GP, Black M, et al Diabetic retinopathy at diagnosis of type 2 diabetes in Scotland. Diabetologia. 2012; 55: 2335–2342. 22688348 10.1007/s00125-012-2596-z PMC3411303

[35] Hammes HP, Kerner W, Hofer S, Kordonouri O, Raile K, Holl RW. Diabetic retinopathy in type 1 diabetes—a contemporary analysis of 8,784 patients. Diabetologia. 2011; 54:1977–1984. 10.1007/s00125-011-2198-1 21638132

[36] Navarro J F, Mora C, Macía M, Garca J. Inflammatory parameters are independently associated with urinary albumin in type 2 diabetes mellitus. American Journal of Kidney Diseases. 2003; 42: 53–61. 12830456 10.1016/s0272-6386(03)00408-6

[37] Tapp RJ, Shaw JE, Harper CA, de Courten MP, Balkau B, McCarty DJ, et al The prevalence of and factors associated with diabetic retinopathy in the Australian population. Diabetes Care. 2003; 26:1731–1737. 12766102 10.2337/diacare.26.6.1731

[38] Nagi DK, Pettitt DJ, Bennett PH, Klein R, Knowler WC. Diabetic retinopathy assessed by fundus photography in Pima Indians with impaired glucose tolerance and NIDDM. Diabet Med. 1997; 14:449–456. 9212309 10.1002/(SICI)1096-9136(199706)14:6<449::AID-DIA367>3.0.CO;2-D

[39] Zhang X, Saaddine JB, Chou CF, Cotch MF, Saaddine J, Friedman DS. Prevalence of diabetic retinopathy in the United States, 2005–2008. JAMA. 2010; 304: 649–656. 10.1001/jama.2010.1111 20699456 PMC2945293

[40] Collard CD, Vakeva A, Morrissey MA, Agah A, Rollins SA, Reenstra WR, etal Complement activation after oxidative stress:role of the lectin complement pathway. Am J Pathol. 2000; 156:1549–1556. 10793066 10.1016/S0002-9440(10)65026-2 PMC1876913

[41] Jack DL, Read RC, Tenner AJ, Frosch M, Turner MW, Klein NJ. Mannose binding lectin regulates the inflammatory response of human professional phagocytes to Neisseria meningitides serogroup B. J Infect Dis. 2001;184:1152–1162. 11598838 10.1086/323803

[42] Fortpied J, Vertommen D, Van Schaftingen E. Binding of mannose-binding lectin to fructosamines: a potential link between hyperglycaemia and complement activation in diabetes. Diabetes Metab Res Rev. 2010; 26:254–260. 10.1002/dmrr.1079 20503257

